# Scoping review of brucellosis in Armenia: persistent neglect, significant knowledge gaps, and a necessity for innovative research, surveillance, and control

**DOI:** 10.3389/fvets.2025.1651077

**Published:** 2025-12-15

**Authors:** Anna Yeritsyan, Christopher G. Laine, Hrant Danelyan, Pertsh Tumanyan, Angela M. Arenas-Gamboa

**Affiliations:** 1 Department of Veterinary Pathobiology, College of Veterinary Medicine & Biomedical Sciences, Texas A&M University, College Station, TX, United States; 2 Reference Laboratory for Especially Dangerous Pathogens of the Republican Veterinary-Sanitary and Phytosanitary Laboratory Services Center, Yerevan, Armenia

**Keywords:** *Brucella*, brucellosis, brucellosis epidemiology, Armenia, one health, neglected zoonotic disease

## Abstract

Brucellosis is a zoonotic disease of nearly worldwide distribution. Despite its endemic presence in many regions, the true burden of the disease remains poorly defined. Armenia, a small country situated in the South Caucasus, exemplifies this lack of clarity. To better understand the epidemiological landscape of brucellosis in Armenia, we conducted a scoping review of studies addressing both human and animal brucellosis within the country. Comprehensive database and manual searches identified only 19 relevant articles published between 1982 and 2023. Reported individual seroprevalence estimates for animal brucellosis ranged dramatically from 0.26 to 18.04% (median—3.33%) in cattle and from 0.75 to 29.90% (median—1.94%) in small ruminants. Only one study investigated swine; however, the sample size was too small and precluded meaningful interpretation. None of the studies identified the *Brucella* species or strains involved, leaving unresolved the question of the primary livestock reservoirs. In humans, brucellosis was predominantly reported among individuals residing in rural areas, with an average incidence of approximately 8 cases per 100,000 population per year. Although constrained by limitations due to the scarcity of available data, this review highlights the neglected nature of brucellosis in Armenia underscoring the potential public health risk posed by this bacterium. Reporting inconsistencies, the absence of readily available diagnostic tools, and the lack of standardized testing procedures hinder an accurate assessment of the disease burden. This review summarizes and identifies critical gaps in research and control efforts, offering insight that can support the need for a comprehensive, long-term national surveillance and control strategy.

## 1 Introduction

Brucellosis, a bacterial zoonosis, is practically globally distributed and significantly impacts the health of both human and livestock populations, as well as the economies that depend on them (1, 2). Among the various species within the genus *Brucella*, three are recognized for their high virulence in their preferred livestock host and their pathogenicity in humans. These include *Brucella abortus*, which primarily affects cattle; *Brucella melitensis*, which infects goats and sheep; and *Brucella suis*, which predominantly infects swine (3, 4).

In animals, *Brucella* is typically transmitted through ingestion or inhalation of infected materials, or through direct contact with contaminated reproductive tissues (5). Infection in livestock can lead to abortion, stillbirth, weak offspring, and reduced milk production (6), resulting in long-term adverse effects on herd productivity and significant economic losses for farmers (2, 7). Human brucellosis is a major global public health concern, with recent models estimating a conservative global incidence of 2.1 million new cases per year, potentially rising to as high as 7.5 million, with the highest risk of infection occurring in Africa and Asia (1, 8). People typically become infected by consuming contaminated unpasteurized dairy products (9, 10) or through direct contact with infected animal reproductive waste (10, 11). Acute symptoms of human brucellosis can present as a flu-like illness characterized by fever, sweats, weakness, and malaise (9). These symptoms closely resemble those of other acute febrile illnesses, such as malaria, typhoid, and influenza, leading healthcare professionals worldwide to frequently misdiagnose brucellosis as one of these diseases (8, 12). If untreated, the disease may progress to a subacute or chronic phase, with possible debilitating long-term sequelae including osteoarticular, gastrointestinal, respiratory, cardiovascular, and neurologic complications (13–15). These complications are also nonspecific and can occur during the course of other diseases, commonly leading to prolonged delays in diagnosis and treatment (16).

Among endemic countries worldwide, insufficient resources, education, and infrastructure have created significant gaps in diagnostics, surveillance, and control of this disease for both livestock and human populations. Armenia, a small country situated in the South Caucasus region of Western Asia, serves as a classic example of a resource-limited country, where the nature and extent of the disease in livestock and humans are largely unknown (17). The presence of seropositive livestock within the country (18), along with all bordering countries having reported to be endemic (19–22), suggest the presence of *Brucella* in Armenia (23); however, the nature and extent of the disease is largely unknown. In light of existing information gaps, the objective of this study is to provide a comprehensive assessment of the extent of disease spread within the country and to identify policy measures and intervention strategies that could be strengthened to enhance disease control. Guided by the framework proposed by Munn et al. (24), we conducted a scoping review, which is particularly well-suited for providing a comprehensive overview of the existing evidence and identifying gaps within the limited knowledge base. This review compiles publicly available information, including (1) scientific research findings, (2) governmental data, and (3) local project activities and results related to the disease and its control. Our findings confirm severe disease neglect, evidenced by (1) significant reporting inconsistencies, (2) the absence of appropriate diagnostic tools in surveillance and research, and (3) deficiencies in the utilized testing procedures, including a lack of standardization, and validation, which prevent us from making any conclusions regarding the disease burden. This review represents an attempt to eliminate assumptions and actively identify and elucidate specific information gaps, as well as to understand the nature of these gaps to guide future investigative efforts. We expect that a better understanding of current challenges and needs will enable policymakers to make informed, data-driven decisions regarding brucellosis surveillance and control, thereby reducing the burden and economic impact of the disease.

## 2 Country profile and livestock systems

**Figure 1:**
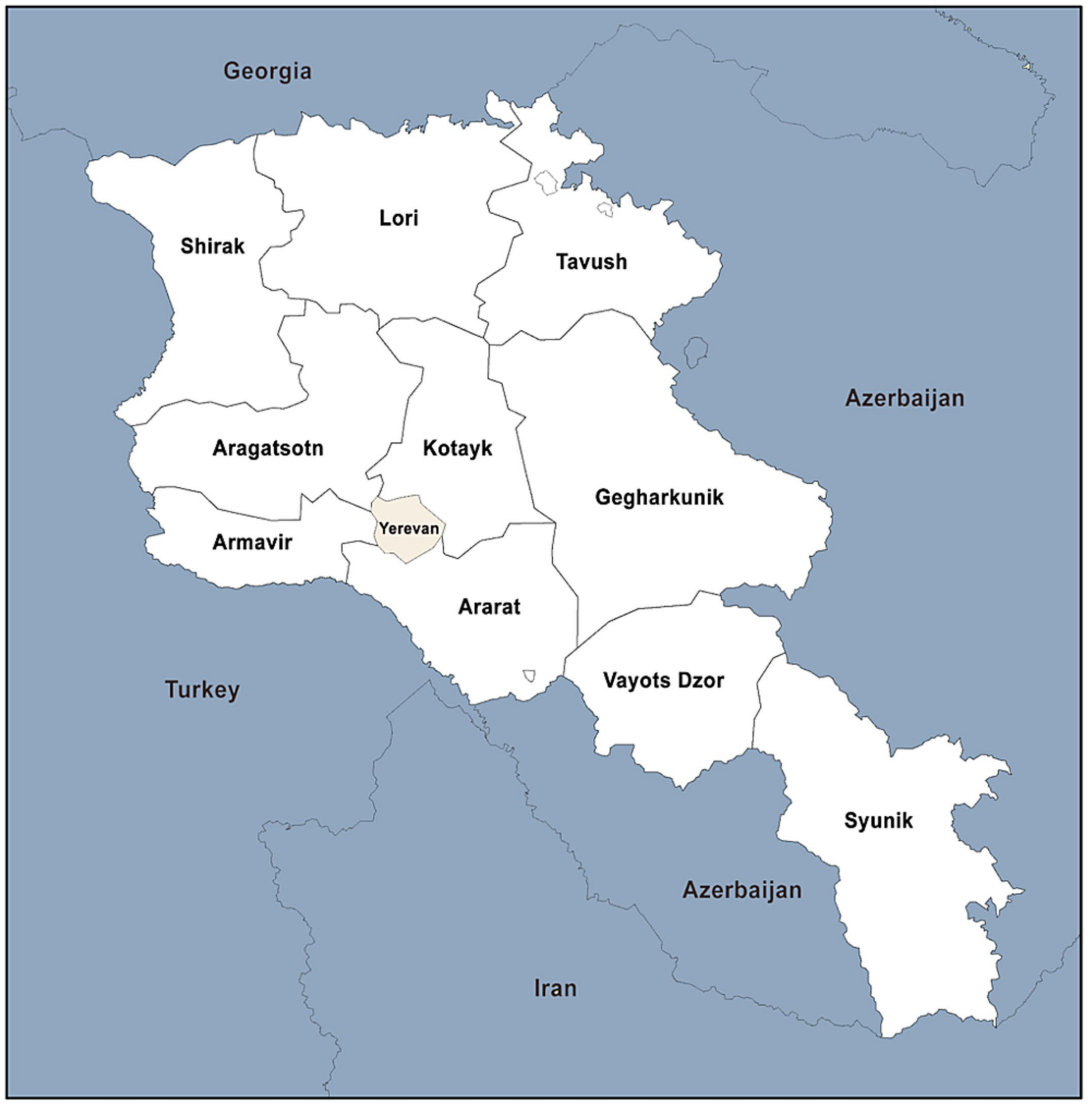
Geographic location of Armenia and its provinces. *Geographic location of Armenia and its administrative subdivisions, including the capital Yerevan and 10 provinces.*

Armenia is a small, landlocked country in the South Caucasus, positioned at the crossroads of Western Asia and Eastern Europe. The country shares borders with Georgia to the north, Iran to the south, Turkey to the west, and Azerbaijan to the east and southwest. There are 11 administrative districts, including the capital, Yerevan, and 10 provinces (Aragatsotn, Ararat, Armavir, Gegharkunik, Kotayk, Lori, Shirak, Syunik, Tavush, and Vayots Dzor) (Figure 1).

The climate can be described as continental with distinct seasons. Since most of the country is more than 1,000 m above sea level, winters in the highlands (in the north and northwest of the country) have an average temperature of −6 °C in January (25). During summer (in the south and southwest) highs can reach +42 °C in July (25). Because of this variability in terrain and climate, regions within the country may have differences in agricultural practices, including crop cultivation and livestock breeding.

Agriculture is one of the most important sectors of the economy, employing about 30% of the country’s workforce (26). The total agricultural output in 2022 was around USD 2 billion (27), with a nearly equal split between crop production and animal husbandry (28). According to the Republic of Armenia (RA) Statistical Committee in 2022, there were approximately 713.7 thousand small ruminants, 559.6 thousand cattle, and 166.1 thousand pigs (29). One notable characteristic is the prevalence of small family farms with an average land size of 1.4 hectares (30). More than 94% of the agricultural output is generated by these small farms with only 6% attributed to commercial farming (29).

The main livestock production systems can be described as pastoral and sedentary (31). Under both systems, there is a lack of livestock movement control measures in place (32). There are public pasture and grassland usage regulations that simply outline rental terms and procedures but do not directly define and enforce safe herd management and pasture usage practices (32). Additionally, there is limited focus on livestock movement across borders; however, it was documented that communities adjacent to the western border had a higher number of brucellosis cases (33).

**Figure 2:**
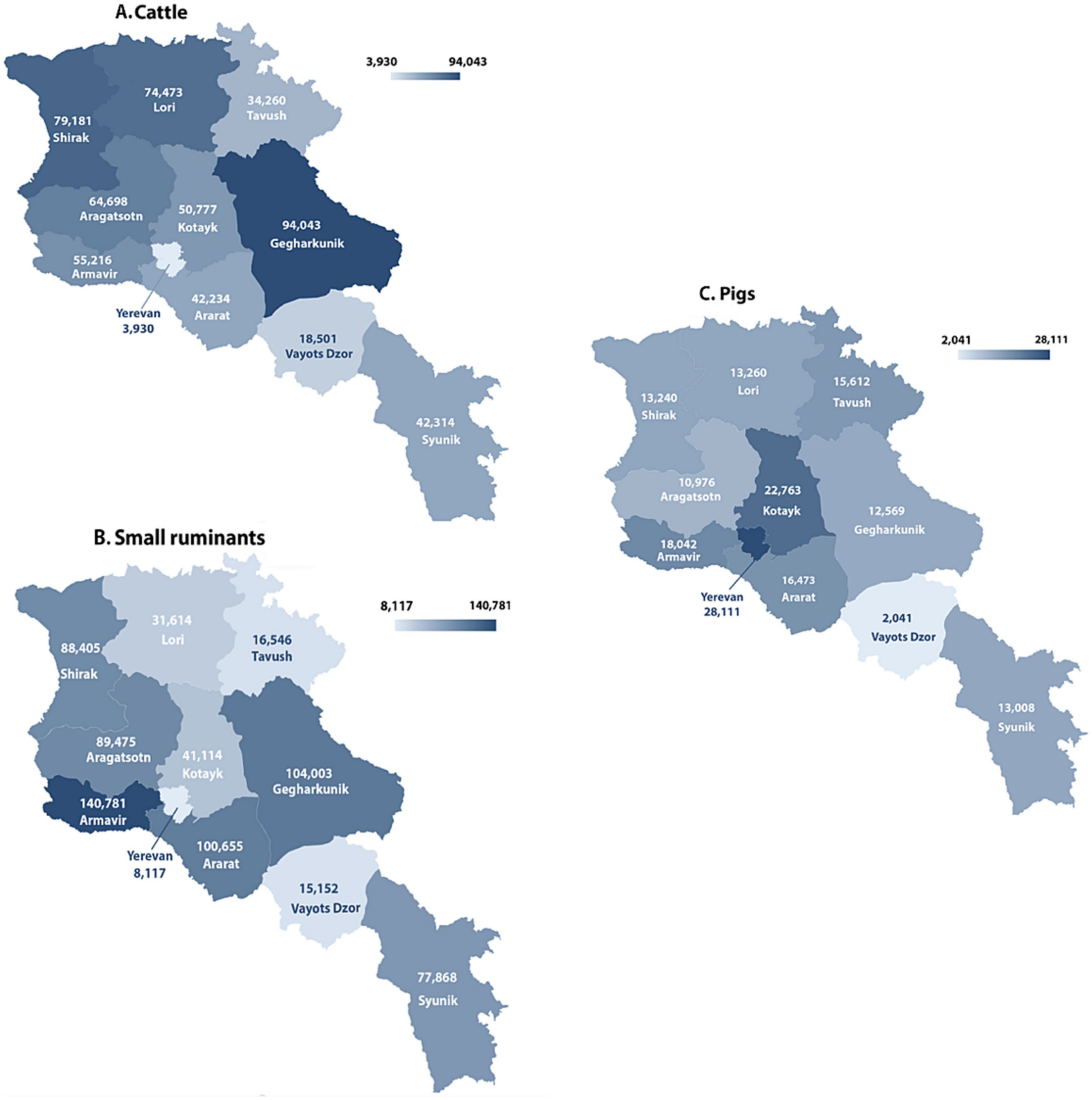
Livestock distribution in 2022 by provinces for **(A)** cattle, **(B)** small ruminants, **(C)** swine. Data extracted from RA Statistical Committee website.

Cattle production is the leading branch of the livestock sector, engaging about 170,000 rural households and rural cooperative farms (34). The cattle market produces about 95% of the milk and almost 58% of the meat in the country (34). Over 90% of cattle are bred by family farms that own fewer than 20 head (18). The largest population of cattle is concentrated in the Gegharkunik, Shirak, and Lori provinces (Figure 2A). Since farmers mainly use the pasture-stall milk production model, there is a very high seasonality in yields, with most of the output received during the summer season.

Small ruminant production is the second most important branch of the livestock sector, primarily conducted on medium to large scales, with about 45% of animals bred by farms that have 50 or more head (18). Sheep and goat breeding is more developed in the sub-mountainous and mountainous areas with extensive pastures, with the largest population of small ruminants bred in the Armavir, Gegharkunik, and Ararat provinces (Figure 2B). During the last decade, the Syunik province expanded small-ruminant production due to its proximity to the border with Iran (the largest exporter of sheep from Armenia). Pig production is also a significant branch of the livestock sector, predominantly carried out by small farms, with over 50% of animals bred by farms that have fewer than 20 head (18). This branch is most developed in Yerevan and the Kotayk province (Figure 2C). In the north, particularly in Tavush and Lori provinces, the mountain-forest ranging of pigs is common (34).

### 2.1 Meat and dairy processing

In 2021, dairy production provided 7.7 million liters of milk and 24.5 thousand tons of cheese, while meat production amounted to about 100 thousand tons (35). There were 150 milk processing plants, 20 abattoirs, and 74 meat processing entities (36). All these production systems contribute to food security, ensuring self-sufficiency of about 90% in beef, 77% in milk, 100% in mutton and 47% in pork consumption.

## 3 Methods

The literature search was implemented in accordance with the Preferred Reporting Items for Systematic Review and Meta-Analyses (PRISMA) guidelines (37). The PRISMA Extension for Scoping Reviews (PRISMA-ScR) checklist has been provided as Supplementary material to enhance transparency and methodological rigor. Major medical and animal disease research databases—including PubMed, CAB Abstracts, Agricola, Gideon, and the Pan-Armenian Digital Library—were systematically searched by the first author on September 16, 2023, for relevant publications. Keywords for the search were (“Armenia” AND “Brucellosis”) OR (“Armenia” AND “*Brucella”*). To mitigate language bias and to capture most of the scientific literature, the search was conducted in English, Armenian, and Russian languages with no limitation on publication year or publication type (peer-reviewed articles, conference presentations, or gray literature). Snowballing—reviewing references from identified articles—was subsequently implemented to mitigate bias introduced by the limited number of databases searched. Finally, to enhance the coverage of literature, a manual search was conducted within the Agriscience and Technology journal published by the Armenian National Agricultural University (ANAU). Online archives of this journal are available for the years from 2019 to 2023 (38).

As the included studies were written in different languages, the study search, inclusion, and data extraction were conducted by the first author only. To mitigate reviewer bias, descriptive summaries of included articles were translated into English and reviewed by the second author. To gain a comprehensive understanding of the situation, inclusion criteria in the full text screening stage were very broad with no limitations based on the quality of publication, methods used, or conclusions. Studies were excluded only if they focused on countries other than Armenia and did not directly pertain to brucellosis incidence. The scoping review protocol is presented in the Supplementary materials.

To further comprehend the brucellosis situation, supporting documents on legislation concerning animal diseases and regulations on food safety were also included. Official statistics on animal production and brucellosis cases in animals and humans were retrieved from the Armenian National Statistical Committee website (39).

## 4 Results from identified studies

**Figure 3:**
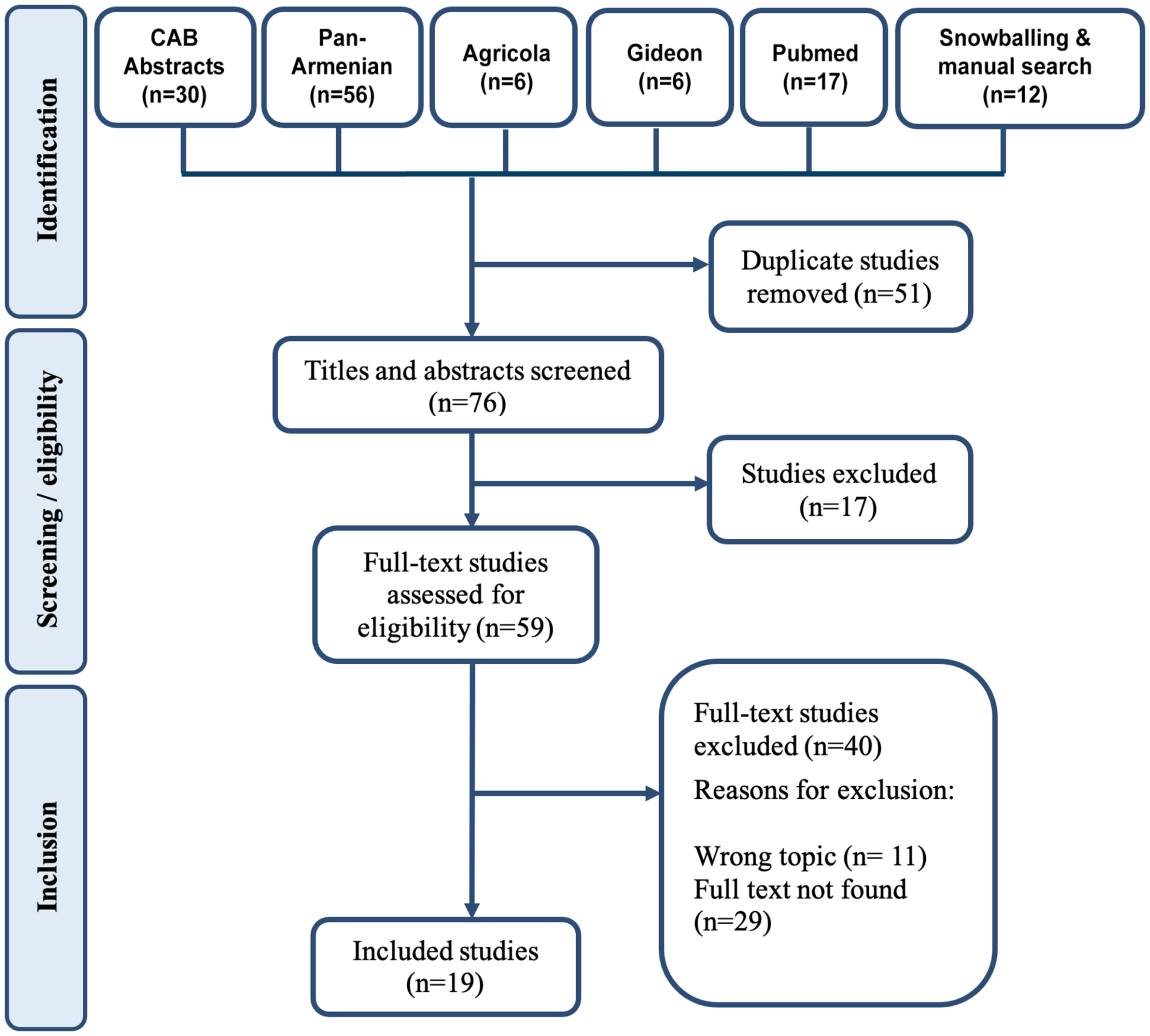
PRISMA diagram for the studies included in the review.

Based on the search terms, 127 articles were identified, and 19 articles were included in this study (Figure 3).

**Figure 4:**
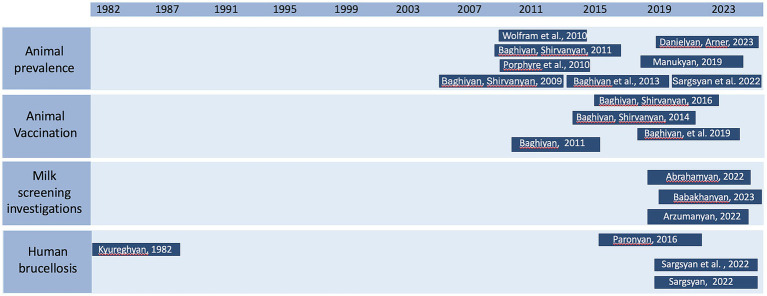
Brucellosis studies conducted in Armenia by topic and publication year.

These articles were published between 1982 and 2023 and studied various aspects. Of these, 12 (63.2%) focused on livestock (eight investigated prevalence, four impact of vaccination on brucellosis incidence in animals), three (15.8%) examined milk quality and the presence of *Brucella* antibodies in milk, and four (21.1%) studied human brucellosis. There was a significant 27-year publication gap between 1982 and 2009, which most likely resulted from the economic hardship experienced after the collapse of the Soviet Union (40). Research resumed in 2009, with eight of the studies (42.1%) conducted in the past 10 years (Figure 4). Additionally, 7 out of 19 publications (36.8%)—all published before 2016—appeared in non–peer-reviewed journals.

### 4.1 Animal brucellosis studies

Livestock brucellosis studies were reported to evaluate the disease burden among primary hosts and to collect additional information, such as risk factors and geographical influences on transmission. Studies were divided into three distinct groups with the objectives of: (1) determining seroprevalence at either the herd or animal level, (2) assessing milk for the presence of antibodies against *Brucella* spp., and (3) evaluating livestock vaccination as a control mechanism.

#### 4.1.1 Livestock prevalence studies

To understand the current brucellosis situation in domestic livestock, we began by assessing the disease burden in primary hosts (i.e., cattle, small ruminants, and pigs). We attempted to review literature that would identify circulating *Brucella* species and provide associated prevalence data. Unfortunately, no studies in the country have been conducted to determine the circulating bacterial species using either bacterial culture or molecular methods (e.g., PCR or next-generation sequencing). The eight articles related to livestock prevalence were limited to serology (seroprevalence studies), which only measure antibodies in the serum, demonstrating potential livestock exposure. Of these studies, one (12.5%) focused on herd seroprevalence (proportion of herds within a defined population that contain at least one seropositive animal), while seven (87.5%) addressed individual seroprevalence (proportion of individual animals within a population that are seropositive) (Table 1).

**Table 1:** Overall findings assessing brucellosis prevalence in livestock in Armenia.

Year	Province	Sampling strategy	Diagnostic tests	Target type	Reported cases	Sample size	Prevalence type	Seroprevalence	Ref
2006–2007	All provinces	All communities	RBT, CFT	Cattle	249 herds	858 herds	Herd prevalence	29.02%	(33)
				Small ruminants	168 herds	858 herds	Herd prevalence	19.58%	
2006–2010	Aragatsotn, Gegharkunk, Kotayk, Syunik, and Armavir	All communities with seropositive animals	RBT	Cattle	2006 = 212; 2010 = 296	2006 = 5,443; 2010 = 10,868	Individual prevalence	2006 = 3.89%; 2010 = 2.72%	(41)
				Small ruminants	2006 = 19; 2010 = 33	2006 = 2,538; 2010 = 3,838	Individual prevalence	2006 = 0.75%; 2010 = 0.86%	
2008	All provinces	All communities with seropositive animals	RBT, ELISA	Cattle	120	11,135	Individual prevalence	1.08%	(42)
				Sheep	58	4,239	Individual prevalence	1.37%	
				Goats	14	558	Individual prevalence	2.51%	
2008	All provinces	All communities	N/A	Cattle	1,618	618,226	Individual prevalence	0.26%	(43)
2009	Shirak, Syunik, and Kotayk	All communities with seropositive animals	RBT and either CFT, SAT, or ELISA	Cattle	345	1,914	Individual prevalence	18.04%	(44)
2010–2014	All provinces	All communities with seropositive animals	N/A	Cattle	8,354	N/A	Individual prevalence	N/A	(45)
2020	Tavush and Kotayk	All accessible regions in the two provinces	RBT, CFT	Cattle	69	472	Individual prevalence	14.62%	(46)
				Sheep	247	826	Individual prevalence	29.90%	
2021–2022	Aragatsotn	One community	RBT, SAT	Swine	5	200	Individual prevalence	2.5%	(47)

1. A common sampling strategy was to select all communities that recorded at least one seropositive animal during previous year national test-and-slaughter control program. 2. Rose Bengal test (RBT), serum agglutination test (SAT), complement fixation test (CFT), enzyme-linked immunosorbent assay (ELISA), not available in the study (N/A). 3. In Armenia, the most common cattle breed is Brown Caucasian; for sheep, it is Armenian Semicoarsewool; and for goats, the predominant breed is Bezoar. Samples for testing are typically collected from reproductive-age animals, regardless of breed. However, the sampling strategies and seroprevalence reported in the studies did not indicate any breed-specific differences. 4. An animal is considered seropositive if all the diagnostic tests applied yield positive results. 5. Seroprevalence estimates are calculated as a ratio of positive cases and sample size provided by the studies. To have more robust conclusions and to avoid statistical bias, results from the studies with smaller sample size should be interpreted with caution.

The single study that addressed herd prevalence was done across all 10 provinces between 2006 and 2007 evaluating the status in both cattle and small ruminants (33) (Table 1). This study utilized the Rose Bengal Test (RBT) followed by the Complement Fixation Test (CFT) only on positive samples to determine seropositivity (33). Out of 858 cattle herds and 858 small ruminant herds tested, 29.02 and 19.58%, respectively, contained at least one seropositive animal (33) (Table 1). Additionally, the study reported an uneven distribution of seropositive herds across the country. Seropositive hotspots for cattle were identified in Aragatsotn, Armavir, and Syunik, while hotspots for small ruminants were found in Ararat and Armavir (33). Tavush was reported to have the fewest communities with infected cattle or small ruminants.

Seven studies investigated the individual prevalence in different livestock species between 2006 and 2022 (Table 1). Of these studies, five (71.4%) occurred from 2006 to 2014 (41–45), and only two (28.6%) were conducted in the last decade (2020–2022) (46, 47). Three studies were conducted on cattle and small ruminants (42.9%) (41, 42, 46), three were conducted solely on cattle (42.9%) (43–45), and one solely on swine (14.3%) (47) (Table 1). No study addressed small ruminants alone. The studies varied in their sampling areas, with only three (42.9%) covering the entire country (42, 43, 45) and four (57.1%) focusing on one (47) or multiple individual provinces (41, 44, 46) (Table 1). From the seven studies, only 5 (71.4%) reported the type of diagnostic testing performed. All five studies assessed seroprevalence using the Rose Bengal test (RBT). In one of these studies, RBT was the sole diagnostic test employed (41). In the remaining studies, RBT was followed by additional diagnostic tests. These tests included the Complement Fixation Test (CFT) (46), the Serum Agglutination Test (SAT) (47), and the Enzyme-Linked Immunosorbent Assay (ELISA) (42). In one study, the choice of follow-up test (CFT, ELISA, or SAT) was determined by reagent availability (44).

Individual seroprevalences reported in cattle across different regions were: 3.89% in 2006 (41), 2.72% in 2010 (41), 0.26% in 2008 (43), 1.08% in 2008 (42), and 18.04% in 2009 (44). The results showed extreme variations within years and provinces, and even within the same province in the same year. For example, studies conducted in all 10 provinces found cattle seropositivity ranging from 0.26 to 1.08% in 2008 (Table 1). Studies involving cattle in Syunik varied from 3.89% in 2006, to 1.05 and 18.04% in 2009, to 2.72% in 2010, and to 0.45% in 2014 (Table 1). Deriving results between timepoints and locations was hindered by the grouping of geographically separate provinces without separating seroprevalence estimates and the degree of fluctuation among individual seroprevalence at specific times and places. For example, the study conducted based on data from 2009 found cattle seroprevalence to be 18.04% (44); however, the investigation only mentions selected communities from different regions without referencing representative samples. (Figure 1).

Individual seroprevalence estimates in small ruminants varied over time, rising from 0.75% in 2006, to 0.86% in 2010 within the same provinces (41). In 2008, seroprevalence was 1.37% in sheep and 2.51% in goats (42) across the entire country. By 2020, the estimate had increased significantly to 29.90% in Kotayk and Tavush (46). Just like in cattle, there were large year-to-year variations within the same province. For example, studies involving sheep in Kotayk reported prevalence estimates ranging from 0.75% in 2006 and 0.86% in 2010, to 29.90% in 2020 (Table 1).

The only study that assessed swine (47) was conducted with a limited number of 200 animals, finding 5 seropositive animals, which is too small of a sample size to accurately estimate seroprevalence (Table 1).

Overall, Kotayk (45, 46), Aragatsotn (42, 43, 45), and Armavir (42) consistently appear as high-prevalence regions across multiple studies. Tavush (42, 43, 45, 46) often reports very low or no cases.

#### 4.1.2 *Brucella* antibodies in milk studies

Dairy is a fundamental part of the Armenian diet. Despite the requirement for commercial dairy producers to pasteurize milk for food safety, there is a substantial informal market where small-scale cheese and milk producers sell unpasteurized dairy products at open-air markets. To evaluate the public health risk posed by this milk, three studies examined the presence of *Brucella* antibodies in cow milk samples collected from these markets across various regions of the country between 2021 and 2022 (Table 2). All three studies employed the Milk Ring Test (MRT). Currently, no research has been conducted on milk from small ruminants.

**Table 2:** Main findings from Milk Ring Test screening investigations.

Year	Province	Sampling strategy	Number positive	Sample size	Percent positive	Ref
2021	Syunik	N/A (selected communities)	0	34	0.00%	(48)
	Ararat		4	34	11.76%	
	Gegharkunik		5	34	14.70%	
	Kotayk		0	34	0.00%	
	Armavir		7	34	20.59%	
	Aragatsotn		0	34	0.00%	
2021	Yerevan	N/A (selected areas in the city)	0	20	0.00%	(49)
2022	Yerevan, Kotayk, Aragatsotn, Gegharkunik	N/A (selected communities)	3	16	18.75%	(50)

The first study conducted in 2021 collected 34 random samples from six regions (48), totaling 204 samples (Table 2). Although the sample sizes are too small to statistically estimate prevalence in each province, a total of 16 samples (7.8%) tested positive for *Brucella* antibodies. Positive samples were found in Ararat, Gegharkunik, and Armavir, while samples from Syunik, Kotayk, and Aragatsotn were all negative (Table 2). The second study conducted in 2021 (49) collected 20 random samples in Yerevan, all of which were negative by MRT (Table 2). The last study done in 2023 (50) collected a total of 16 random samples across four regions (Table 2). One sample was from Yerevan, six from Kotayk, six from Aragatsotn, and three from Gegharkunik. The results showed no positive samples in Yerevan and Kotayk; however, one sample from Gegharkunik and two from Aragatsotn tested positive (Table 2).

#### 4.1.3 Impact of vaccination on brucellosis incidence in animals

While vaccination is a globally recognized cornerstone of brucellosis control, Armenia’s efforts have been inconsistent. Currently, vaccination is not implemented as an official control strategy against brucellosis. Historically, vaccination with N19 or N82 for cattle and with Rev-1 for small ruminants began in 1953 but was discontinued following the collapse of the Soviet Union in 1990 (51). Vaccination was reintroduced as a pilot program from 2009 to 2014, focusing on Syunik province, although the rationale for selecting this region was not explained. The program used RB-51 to vaccinate heifers aged 4–12 months and Rev1 (subcutaneous administration) to vaccinate female lambs and kids aged 3–8 months (52). Despite plans for expansion, limited funding prevented the program from scaling beyond this initial phase. The pilot program led to four studies covering the period from 2007 to 2014. These studies aimed to demonstrate the decrease in the number of seropositive animals before and after vaccination; however, they did not consistently report the total number of animals in each included community, limiting the ability to calculate and compare prevalence data (see Table 3).

**Table 3:** Main findings from research studies on effect of vaccination.

Year	Province	Sampling strategy	Diagnostic tests	Target type	Time point	Reported cases	Ref
2007–2017	Syunik	All communities with vaccinated animals	RBT, SAT	Cattle	Pre-vaccination	317	(51)
					Post-vaccination	96	
				Small ruminants	Pre-vaccination	246	
					Pre-vaccination	134	
2009–2010	Syunik	All regions with vaccinated animals	N/A	Cattle	Pre-vaccination	334	(52)^a^
					Post-vaccination	166	
2009–2014	Syunik	All regions with vaccinated animals	RBT, ELISA	Cattle	Pre-vaccination	211	(53)
					Post-vaccination	50	
				Small ruminants	Pre-vaccination	240	
					Post-vaccination	26	
2009–2014	Syunik	N/A (Selected communities)	RBT, SAT	Cattle	Pre-vaccination	456	(40)
					Post-vaccination	67	
				Small ruminants	Pre-vaccination	321	
					Post-vaccination	26	

1. Rose Bengal test (RBT), serum agglutination test (SAT), enzyme-linked immunosorbent assay (ELISA), not available in the study (N/A). 2. In Armenia, the most common cattle breed is Brown Caucasian; for sheep, it is Armenian Semicoarsewool; and for goats, the predominant breed is Bezoar. Samples for testing are typically collected from reproductive-age animals, regardless of breed. However, the sampling strategies and seroprevalence reported in the studies did not indicate any breed-specific differences. 3. An animal is considered seropositive if all the diagnostic tests applied yield positive results.

a Study discusses small-ruminant vaccination as well, but specific pre- and post-vaccination cases for small ruminants are not presented.

The study region was Syunik province, and the research timeframe was 2009–2014, with one study extending it until 2017 (51). The diagnostic assays used to determine seroprevalence in two studies (50%) were RBT and SAT (40, 51), and in one study (25%) RBT and ELISA (53). One study (25%) did not mention the diagnostic method (52). Three studies (75%) addressed vaccination in both cattle and small ruminants (40, 51, 53) and one (25%) focused exclusively on cattle (52). Studies on cattle reported a decline in number of seropositive animals before and after vaccination from 317 to 96 (51), from 344 to 166 (52), from 211 to 50 (53), and from 456 to 67 (40). Studies on small ruminants reported a decline in number of seropositive animals before and after vaccination from 246 to 134 (51), from 240 to 26 (53), and from 321 to 26 (40). The total number of animals tested before and after vaccination was reported only in the last study (40).

### 4.2 Humans brucellosis studies

Human brucellosis is closely linked to brucellosis in animals. We reviewed the literature to understand the current situation of human brucellosis by identifying circulating *Brucella* species and providing prevalence data. Unfortunately, no studies have been conducted in the country that demonstrate organism isolation or species determination using bacterial culture or molecular methods (e.g., PCR or next-generation sequencing) in humans.

Four articles on human brucellosis, published between 1982 and 2019, were identified. All were limited to describing seroprevalence (Table 4). One study (25.0%) focused on seroprevalence among abattoir workers (54), two (50.0%) reported incidence in the general population (55, 56), and one (25.0%) assessed febrile hospital patients (57) (Table 4).

**Table 4:** Main findings from studies on human brucellosis.

Year	Province	Sampling strategy	Target type	Diagnostic tests	Reported cases	Sample size	Seroprevalence	Ref
1982	Shirak	N/A	Abattoir/meat packing plant employees	W&H	80	697	11.48%	(54)
2004–2014	All 10 provinces and Yerevan	All humans brucellosis cases reported by the State	General population	N/A	Average = 7.95 (per 100,000); Minimum = 0.06 (Yerevan); Maximum = 95.3 (Vayots Dzor)	N/A	N/A	(55)
2016–2019					Average = 8.13 (per 100,000); Minimum = 0.4 (Tavush); Maximum = 33.7 (Syunik)			
2010–2012	Yerevan	All febrile patients in a medical center	Hospital patients	W&H and RBT	50	600 febrile patients	N/A	(57)
2016–2019	All 10 provinces	All animal and human brucellosis cases reported by the State	General population	N/A	1,006	N/A	N/A	(56)

Rose Bengal test (RBT), Wright and Huddleston (W&H), not available in the study (N/A).

The first study (54), conducted in Shirak in 1982, confirmed clinical manifestations of brucellosis. Using Wright and Huddleston’s tests, the investigators identified 80 exposed abattoir workers (54). Symptoms included joint pain, fatigue, and sleep disorders (54). Additionally, 21 patients reported fever and 39 reported sciatica. The nervous system was affected in six employees (54). The study concluded that the frequency of infection is related to occupational tasks, with risks increasing with the duration of work (54).

The next study (55) analyzed data reported by the National Center for Disease Control (NCDC) from 2004 to 2014 and from 2016 to 2019 to define epidemiological zones based on brucellosis incidence (Table 4). This study covered all regions of the country, although it did not provide information on the diagnostic tools used (55). The findings indicated an average annual incidence of 7.95 new cases of brucellosis per 100,000 population from 2004–2014 (55) (Table 4). The highest incidence was in Vayots Dzor (95.3 per 100,000), while the lowest was in Yerevan (0.06 per 100,000). Similarly, from 2016–2019, the average annual incidence was 8.13 new cases per 100,000 population, with the highest in Syunik (33.7 per 100,000) and lowest in Tavush (0.4 per 100,000) (55) (Table 4). Based on the percentiles of new reported cases by region, the authors concluded that Vayots Dzor, Shirak, and Armavir provinces are at the highest risk.

The next study (57) examined common causes of zoonotic infections and analyzed the association between febrile illness and brucellosis in patients hospitalized with febrile illness symptoms from 2010 to 2012 (Table 4). Patients were from Yerevan, Aragatsotn, Gegharkunik, and Kotayk. Serologic assays used for identifying brucellosis included RBT and Wright & Huddleston tests. Out of 600 patients with febrile illness symptoms, 50 (8.3%) were diagnosed with brucellosis (57) (Table 4). The main clinical symptoms observed were excessive sweating, joint pain, and muscle soreness (57). Furthermore, the study revealed that 76% of patients had a history of consuming raw milk, and nearly one-fourth of the brucellosis patients had contact with animal aborted materials or were involved in animal slaughter (57).

The most recent study (56) aimed to conduct a comparative analysis of new brucellosis cases among humans and animals from 2016 to 2019 (Table 4). Data was obtained from the World Organization for Animal Health (WOAH) website and official reports from the NCDC; however, no information was provided on the diagnostic tests used (56). The findings suggest that the ratio of animal to human brucellosis cases increased significantly from 0.18 in 2016 to 16.57 in 2019 (56). Examining individual provinces, the highest ratio of animal to human brucellosis was in Vayots Dzor (39.5%), while Tavush had the lowest ratio, with no human cases reported in 2019 (56).

### 4.3 Brucellosis control, diagnostics, surveillance, and current situation

Brucellosis is considered a priority zoonotic disease by the government. The policy and legal framework for its control is established through (1) Law of the Republic of Armenia on Veterinary Medicine, N137-N, 2014 (Regulation assigning agencies and entities responsible for animal health control and management, defining the roles and responsibilities of involved parties, and including requirements for livestock management, meat and dairy production, and proper slaughter procedures), (2) Order of the Head of the Food Safety Inspection Body (FSIB), N418-N, 2013, July 16 (Instruction for control and prevention of brucellosis), and (3) Order of the Head of FSIB, N422-A, 2014, May 8 (Guideline for the implementation of compulsory slaughter of farm animals in case of brucellosis).

#### 4.3.1 Brucellosis diagnostic and surveillance systems for livestock

The national strategy for controlling brucellosis currently employs a test-and-slaughter method for cattle and small ruminants. According to this policy, regional laboratories under the FSIB test reproductive-age cattle twice a year, while small ruminants are tested once annually (with additional testing implemented in the event of outbreaks). RBT is currently employed as the screening method. Seropositive test results obtained exclusively through RBT from regional laboratories are sent to the Reference Laboratory for Especially Dangerous Pathogens (RLEDP) for additional testing using RBT, CFT, or ELISA (depending on availability). This means that if RBT is the only available reagent at RLEDP, animals are considered positive based solely on RBT results. Positive results are then reported to the Veterinary Department of the FSIB and the associated provincial veterinary inspector. The veterinary inspector notifies livestock owners, who are responsible for slaughtering the infected animals. Slaughter must take place in slaughterhouses, but due to the limited number of operational facilities, home slaughtering for the owner’s consumption is permitted under veterinary supervision. Additionally, farms with a seropositive case are prohibited from selling raw milk or cheese until the farm is clear from infection. Boiled milk from affected farms is safe for general consumption in food. However, it must not be supplied to daycares, schools, and hospitals, where children and immunocompromised patients could be at risk. Under current regulations, farmers bear a substantial financial loss, as there is no compensation for culled animals, and many slaughterhouses may refuse to accept infected livestock. As a consequence, animal owners often choose not to cull infected animals and instead sell them in the market while concealing their health status.

#### 4.3.2 Current situation of livestock brucellosis

**Figure 5:**
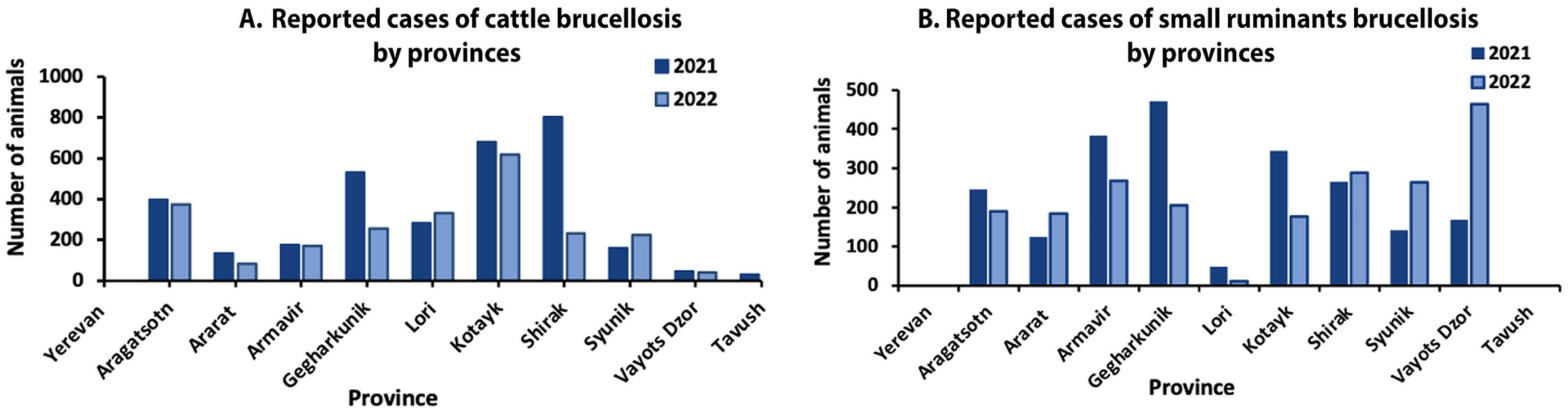
Number of reported brucellosis cases in **(A)** cattle and **(B)** small ruminants in 2021–2022 by provinces (data reported by the RA Statistical Committee).

National surveillance data on livestock brucellosis prevalence based on RBT and other tests (when available) indicate a widespread and uneven distribution of brucellosis among cattle and small ruminants throughout Armenia (Figure 5).

In 2021 and 2022, regions most affected by cattle brucellosis were Kotayk (682 and 617 cases) and Shirak (801 and 232 cases). The lowest number of cases was reported in Tavush (29 and 12), Yerevan (36 and 22), and Vayots Dzor (46 and 42). The official statistics show fluctuations in the number of reported cases between the years. For example, from 2021 to 2022, in cattle, Gegharkunk reported 531 and 256 (a 51.8% reduction) and Shirak reported 801 and 232 (a 71.0% reduction) (Figure 5). In small ruminants, the most affected regions in 2021 and 2022 were Gegharkunik (470 and 205 cases), Armavir (381 and 232 cases), and Vayots Dzor (167 and 463 cases). The least affected regions were Tavush (0 and 9 cases), Yerevan (0 and 11 cases), and Lori (47 and 11 cases). Significant fluctuations between the years were observed in Vayots Dzor, (117.2% increase from 167 to 463), Syunik (87.2% increase from 141 to 264), and Gegharkunk (56.4% reduction from 470 to 205).

By dividing the officially reported number of positive cases by the total number of livestock, we estimated a prevalence of 0.42% in cattle and 0.29% in small ruminants. Interestingly, these prevalence estimates are lower than those reported in the literature, which range from 0.26% to 18.04% for cattle and 0.75% to 29.9% for small ruminants (Table 1, Figure 5).

#### 4.3.3 Brucellosis diagnostic and surveillance systems for humans

The National Center for Disease Control and Prevention (NCDC) is responsible for monitoring and managing human brucellosis cases. Although a concept of One Health adopted in 2015 (58) assumes the collaboration of state agencies involved in human and animal health, practical One Health collaboration is very limited. Human health data reporting from the NCDC to veterinary authorities is limited to information on a patient’s village due to patient data confidentiality issues. Testing procedures are not standardized, as, in addition to a few public health laboratories, there is a large pool of private laboratories that purchase their own diagnostic tests and operate independently.

#### 4.3.4 Current situation of human brucellosis

**Figure 6:**
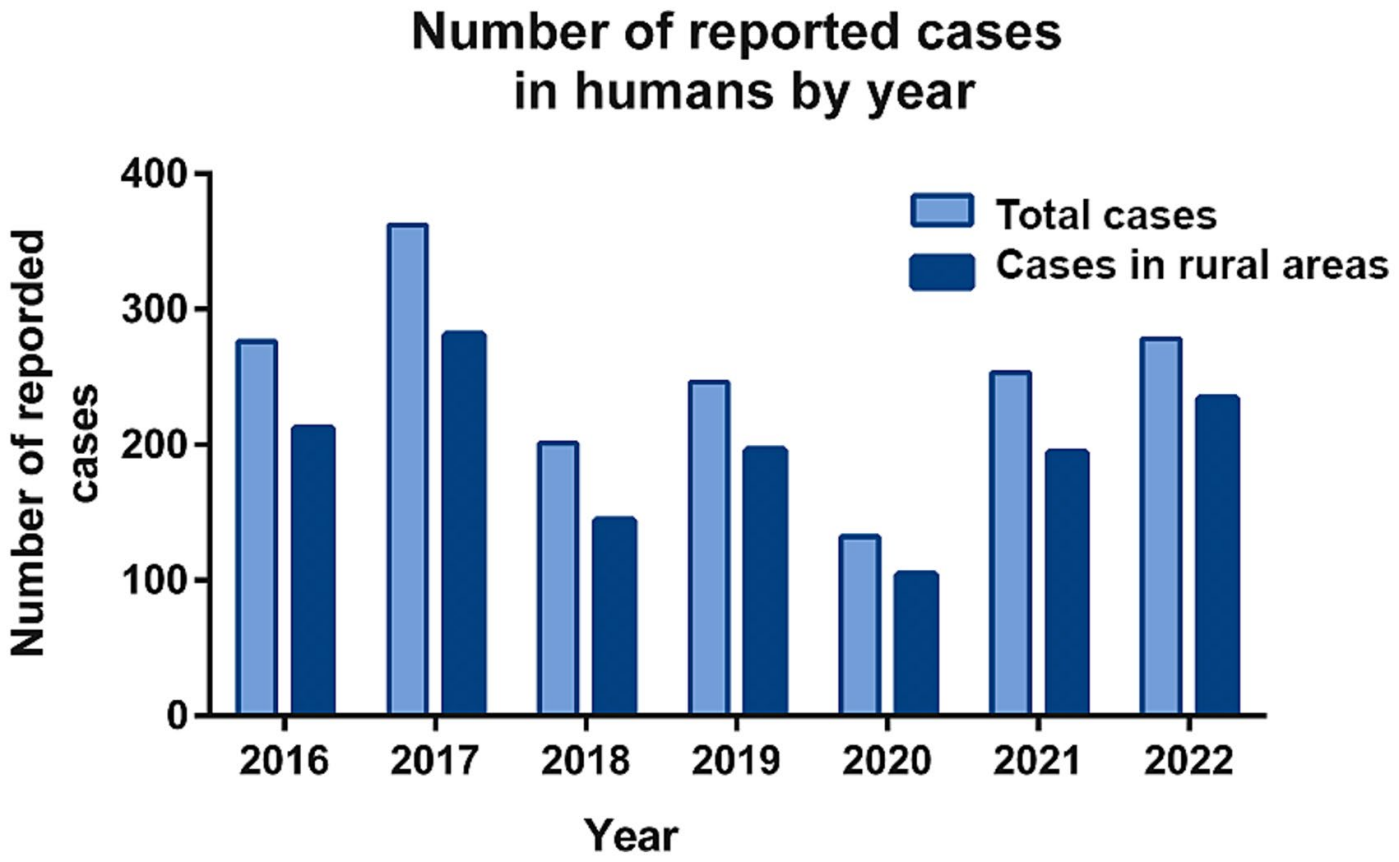
Newly reported cases of human brucellosis from 2016 to 2022.

Official data on new human cases have been reported since 1999, but information on cases in rural areas has only been available since 2016. Recent reports show a consistent upward trend in human brucellosis cases, increasing from 201 (6.7 per 100,000 population) in 2018 to 278 (9.4 per 100,000 population) in 2022 (Figure 6). The exception is 2020, with 132 cases (4.4 per 100,000 population), likely due to underreporting during COVID-19 (Figure 6). Most human cases were concentrated in rural areas, accounting for 78.1% (1,365 out of 1,748) of the total cases (Figure 6).

## 5 Discussion

Brucellosis is a global bacterial zoonosis that significantly affects human and livestock health. *Brucella* has long been recognized as endemic across Asia, making it one of the highest-risk regions for livestock and human brucellosis globally (1). Armenia exemplifies a resource-limited country where the true extent of brucellosis remains unclear. Despite evidence of seropositive livestock and endemic neighboring countries, data gaps hinder understanding the disease prevalence and effectiveness of control efforts. This scoping review was implemented to identify these gaps and to shed light on the extent of the disease burden in the country.

### 5.1 Livestock brucellosis

An analysis of livestock brucellosis prevalence studies conducted between 2006 and 2022 reveals substantial variation across several dimensions: timeframe and geographic coverage, diagnostic methodologies, reported prevalence estimates and regional trends. Most of the studies conducted over this 16-year period were concentrated between 2006 and 2014, with only two studies published in the last few years (2020–2022). This decline in recent research likely reflects reduced surveillance efforts, possibly due to shifting priorities and limited resources. Geographic scope also varied, with fewer than half of the studies covering the entire country. Certain regions, such as Kotayk, Aragatsotn, and Armavir, consistently reported higher prevalence estimates, while others like Tavush and Yerevan, showed very low or no cases. However, underreporting or limited testing in these areas cannot be ruled out. Additionally, significant variation within provinces (41, 43) suggests the presence of localized risk factors, highlighting the need for targeted surveillance and intervention strategies. Factors contributing to high prevalence can be commingling of animals on summer pastures and backyard slaughter (43, 44), delays in separation of sick animals from the herd (42), or purchasing livestock without requesting a serologic examination (45). Disease frequency is positively correlated with community densities and the proportion of Yezidis (national minority) in the community (33). While this variation offers both national and localized perspectives, it complicates cross-study comparisons due to differing sampling strategies and regional focuses. Further complications in comparing research findings arise due to inconsistencies in diagnostic testing procedures. The Rose Bengal Test (RBT) is used for initial screening at the regional laboratories, with positive samples forwarded to the central laboratory in Yerevan for further testing using either RBT, SAT, CFT, or ELISA (44) (Table 1), highlighting the absence of a standardized diagnostic protocol. Some studies did not specify the diagnostic methods used, limiting the interpretability and comparability of their findings. In addition, a study on swine brucellosis (47) used SAT as a secondary test. According to WOAH (59) terrestrial manual, SAT can be used for cattle only, and it is not specific enough to be used for disease confirmation. Overall, the absence of a standardized diagnostic protocol across studies is a major limitation, as it undermines the reliability of prevalence estimates and hampers efforts to monitor disease trends over time. It is important to note that the number of reported cases is closely tied to testing volume; increased testing typically leads to the identification of more cases. Comparing results across studies presents an additional challenge, as none of them referenced the sensitivity or specificity of the diagnostic tests used—information that is essential for generating more accurate estimates of true prevalence. In Armenia, the Rose Bengal Test (RBT) is the primary serological method employed for routine screening. While RBT is widely used due to its simplicity and cost-effectiveness, its diagnostic sensitivity and specificity can vary depending on testing conditions and population characteristics. A meta-analysis by Greiner et al. (60) reported average sensitivity and specificity values of 98.1 and 99.8%, respectively. However, other studies have documented lower performance, with sensitivity as low as 86% and specificity around 88% (61). Since bacterial culturing is not implemented in Armenia, the diagnostic accuracy of locally conducted RBT remains undefined, leaving the true prevalence uncertain.

The detection of *Brucella* antibodies in milk samples from Ararat, Armavir, Gegharkunik, and Aragatsotn indicates a potential health risk for consumers of unpasteurized dairy products in these regions. However, the small sample sizes (ranging from 16 to 204) limit the statistical strength of these findings and restrict their generalizability to the wider population. Interestingly, no positive results were reported in livestock high-prevalence areas such as Kotayk and Syunik. This absence may be attributed to sampling bias, limitations in test sensitivity, or temporal fluctuations in milk contamination levels. Furthermore, the high brucellosis risk areas reported in the research studies do not coincide with the reported numbers from national surveillance (Figure 5). A notable gap in the data is the lack of testing on milk or cheese samples from small ruminants, which is particularly concerning given the recent reports of high seroprevalence in sheep. Although preliminary, these findings raise a credible public health concern regarding the consumption of unpasteurized milk and dairy products and highlight the urgent need for surveys with larger and more representative sample sizes.

Some studies on livestock vaccination have reported a decline in seropositivity among cattle and small ruminants, which may suggest a potential impact of vaccination efforts. However, the absence of key methodological details, such as the total number of animals tested (51, 53), diagnostic assays used (52), and the consistency in geographic distribution of sampling (40), limits the reliability and generalizability of these findings. While preliminary results indicate that the pilot program may have contributed to reduced brucellosis prevalence in livestock, the overall lack of scientific robustness, transparency, and methodological rigor prevents definitive assessments. Serological tests alone are insufficient for evaluating vaccine effectiveness, and reporting seropositive reactors without the total number of samples tested limits the validity of any comparison. For future vaccination initiatives, it is essential to implement comprehensive data collection, adopt standardized diagnostic procedures, and ensure broader geographic coverage. Most importantly, identification of *Brucella* species circulating in the country needs to precede any future vaccination effort. Currently, there is an unofficial ban on the culture and isolation of *Brucella,* which makes it clear that the pilot vaccination was conducted without confirming the appropriate vaccine (whether against *B. melitensis* or *B. abortus*) for targeted animal intervention.

### 5.2 Human brucellosis

The scope of human studies is also fragmented. Some studies focused on occupational exposure among abattoir workers (54), others analyzed national data (55, 56), or assessed febrile patients for zoonotic infections (57). This uneven distribution of research topics leaves major gaps in understanding the full range of human brucellosis risk factors, transmission pathways, and regional patterns. An evaluation of human brucellosis research reveals significant diagnostic and methodological shortcomings, including the absence of standardized diagnostic protocols, no molecular confirmation of infection, small sample sizes or limited geographic representation, and a lack of longitudinal or trend analysis. Despite these limitations, the available evidence suggests that human brucellosis is a significant but underrecognized public health issue. Additionally, the disease appears to be regionally concentrated and associated with livestock exposure and consumption of unpasteurized dairy products (57). Vayots Dzor, Shirak, and Armavir provinces were found to be among high exposure risk provinces for humans (55). Interestingly, Vayots Dzor and Armavir were also considered hotspots for small ruminant brucellosis (33), Shirak had a high cattle prevalence (18.04%, along with Syunik and Kotayk) (44) (Table 1) and Armavir had 20.59% of milk samples testing positive by MRT (48) (Table 2). Thus, controlling zoonotic diseases at their source (within animal populations) can enhance food safety and reduce the risk of transmission to humans.

### 5.3 Control policies

An overall evaluation of Armenia’s brucellosis control efforts reveals that, while the country has a solid legal and policy framework—including relevant laws and decrees—there are notable gaps in implementation and coverage. A key advancement is the national livestock identification system: cattle tagging has been widely adopted since 2021, and tagging of small ruminants began in 2025. However, the exclusion of swine from national policy remains a significant oversight, particularly in light of recent evidence confirming brucellosis in pigs (47). Armenia’s primary control strategy is the test-and-slaughter approach, which is comprehensive in design but faces practical challenges. The main challenge is the lack of compensation for culled animals, aside from partial payments from slaughterhouses. This financial burden discourages farmer compliance and may lead to underreporting or concealment of infected animals. Additionally, limited access to licensed slaughterhouses forces many farmers to resort to home slaughtering, which increases the risk of disease transmission and undermines biosecurity measures. The surveillance system is structured and routine, involving biannual testing of cattle and annual testing of small ruminants, along with mandatory reporting of abortions for laboratory investigation. However, the diagnostic infrastructure is critically underdeveloped. The Rose Bengal Test (RBT) is the primary screening tool, but the antigens used are procured through a state tender process that favors the lowest bidder, without considering reagent quality. Additional tests such as ELISA, FPA, and CFT are used inconsistently and lack internal validation and standardization to local conditions. These diagnostic limitations severely compromise the reliability of test results and hinder the country’s ability to track outbreaks or understand the epidemiology of the disease. Strengthening diagnostic capacity, ensuring quality control, and expanding the scope of surveillance are essential steps toward a more effective brucellosis control program.

### 5.4 A way forward

Armenia must prioritize strengthening its diagnostic capacity and laboratory infrastructure to effectively manage brucellosis. This includes establishing *Brucella* culture, validating serological assays, and utilizing molecular-based approaches when deemed necessary, following WOAH guidelines at both central and regional levels to enable species identification, outbreak tracing, and vaccine validation. It is also crucial to standardize diagnostic protocols across all laboratories using WOAH-recommended tests, such as RBT alongside iELISA or CFT. Implementing robust quality assurance systems for all diagnostic reagents and procedures, including internal validation and the use of control sera, is essential. Additionally, regional laboratories must be properly equipped and staffed to reduce reliance on central facilities and to ensure the integrity of collected samples.

Improvements in surveillance and data management are also necessary. The country should integrate animal and human health surveillance systems to facilitate real-time data sharing and joint analysis. Ensuring consistent, nationwide testing coverage with harmonized sampling strategies will enhance the comparability of data across regions and over time. Surveillance data should include *Brucella* species identification to better understand transmission dynamics and inform targeted control strategies. Furthermore, discrepancies between official and independent data sources must be addressed to improve transparency and reliability.

Expanding One Health coordination is another critical step. This can be achieved by operationalizing existing frameworks through the establishment of joint outbreak investigation teams and formalizing communication protocols between sectors. Regular joint data-sharing and collaborative training exercises between veterinary and public health agencies will help build trust and improve coordination.

A comprehensive national control program must be implemented to replace fragmented interventions. This program should be long-term, fully funded, and inclusive of all livestock species—particularly swine, which are currently excluded from national policy despite evidence of infection. Ensuring access to licensed slaughterhouses and introducing a fair compensation scheme for culled animals will encourage farmer compliance with test-and-slaughter policies. The government should educate farmers and dairy producers on safe animal handling and the importance of milk pasteurization, while also launching public health campaigns in high-risk rural areas to raise awareness of brucellosis symptoms and prevention methods.

Finally, the adoption of an evidence-based vaccination strategy should become a priority. Vaccination programs should not be implemented without first identifying the circulating *Brucella* strains. Campaigns must be designed based on epidemiological data and monitored for effectiveness through standardized, transparent studies. Ensuring vaccine quality through local validation and post-vaccination surveillance is also essential for long-term success.

### 5.5 Study limitations

Given the aim of providing an overview of the brucellosis situation and considering the limited number of studies and the variability in their results, a scoping review is the most appropriate design for this research. However, this design has several limitations. First, studies were selected from a limited number of databases, potentially resulting in incomplete coverage of relevant literature. Additionally, not all included articles were peer-reviewed, which is a key indicator of scientific rigor and reliability. However, to present a comprehensive picture of the situation, studies published in non-peer-reviewed journals were also included, especially given the limited information available on the topic.

## 6 Conclusion

The findings underscore a systemic issue in Armenia’s brucellosis surveillance and research framework. Key recommendations for national surveillance include reducing risks associated with backyard slaughter by improving access to regulated slaughterhouses, providing financial compensation to farmers, encouraging the proper disposal of sick animals, and preventing further disease transmission. In addition, it is important to implement capacity-building of veterinary services and improve data integration between animal and human health sectors to support the One Health approach. The main recommendations for improving research and the diagnostic framework include standardizing diagnostic protocols in line with WOAH guidelines, training laboratory personnel to ensure proper test selection and interpretation, and investing in strain identification to inform targeted vaccination and control strategies. Addressing these issues is essential for building a reliable evidence base that can guide effective brucellosis control and reduce the disease burden.
