# Supplementary material for: Scoping review of brucellosis in Armenia: persistent neglect, significant knowledge gaps, and a necessity for innovative research, surveillance, and control
Source: Front Vet Sci. 2025 Dec 15;12:1651077. doi: 10.3389/fvets.2025.1651077 (PMC12745417; doi:10.3389/fvets.2025.1651077)
Supplement: Supplementary file 2 [file Data_Sheet_2.docx]

**Protocol for**

Scoping review of brucellosis in Armenia: persistent neglect, significant knowledge gaps, and a necessity for innovative research, surveillance, and control

1. **Introduction**

Brucellosis is an infectious zoonic disease that has been recognized by WOAH as the second most important disease after Rabies (Choudhary et al. 2019). Although globally distributed and endemic in the developing world, yet it is often unrecognized and underreported (Laine, 2022) by individual countries. This study focuses on Armenia, a country located in South Caucasus, where brucellosis is known to be present, however the nature and extent of disease remain unknown.

To better understand the disease situation, we conducted a scoping review of studies exploring animal and human brucellosis in the country.

1. **Objective**

In this study, we aim to describe the currently known status of animal and human brucellosis in Armenia as a first step to identify local strengths, gaps and opportunities in the veterinary and public health systems.

This study would better allow policymakers to make informed, data-driven decisions regarding brucellosis surveillance and control, thus decreasing the burden and economic consequences of the disease.

1. **Methods**

Online databases specialized on health and veterinary topics and systematically searched to identify relevant literature are:

- PubmMed,
- Agricola,
- CAB Abstracts,
- Gideon, and
- Pan-Armenian Digital Library

Keywords used in searches should be (Armenia) and (Brucellosis), as well as (Armenia) and (*Brucella*). To cover most of the literature on the topic, search should be implemented in English, Armenian, and Russian languages, with no limitation on publication year or publication type (peer-reviewed articles, conference presentations, gray literature). To maximize number of identified publications, no limitation on time will be introduced. In addition, all references from identified publications should be searched as well.

Manual search from issues of Agriscience and Technology journal published by the Armenian National Agricultural University (ANAU) available online will also be conducted. Snowballing will be implemented to maximize number of articles found and to mitigate bias introduced by the limited number of databases searched.

1. **Study screening and data extraction**

Since database search and extraction requires knowledge of three languages, only one reviewer (the first author) will implement the review. Second author will look through the English summaries of articles to ensure inclusion justification. General information, as well as context and related evidence will be extracted from each study in data extraction stage.

1. **Inclusion/Exclusion criteria (PCC – population, concept, context)**

|  | Inclusion | Exclusion |
| --- | --- | --- |
| P: Population | - Domestic and agricultural animals that are infected with *Brucella* - Humans infected by *Brucella* (whether occupational or infected through food). This also includes studies on milk testing. | - Brucellosis in non-domestic animals and in animals not considered agricultural livestock (wildlife) - Diseases other than Brucellosis |
| C: Concept | - Any interventions including policies and control actions adopted by governments, multilateral, international and national NGOs, to combat brucellosis in animals and humans - Any outcomes resulting from the interventions, including reduction in the disease incidence. | - Simulation studies, willingness to pay, and hypothetical experiment studies. |
| C: Context | - Geographic location, cultural factors or specific settings that provide details on the disease situation. | - Regions other than Armenia |

1. **Ethics and dissemination**

The proposed scoping review will help guide the development of the future brucellosis related actions or research, thus the findings will be disseminated through peer-reviewed publications and conference presentations.
